# Data in support of proteome analysis of gynophores and early swelling pods of peanut (*Arachis hypogaea* L.)

**DOI:** 10.1016/j.dib.2015.11.029

**Published:** 2015-11-25

**Authors:** Han Xia, Nana Jiang, Lei Hou, Ye Zhang, Changsheng Li, Aiqin Li, Chuanzhi Zhao

**Affiliations:** Bio-Tech Research Center, Shandong Academy of Agricultural Sciences, Shandong Provincial Key Laboratory of Crop Genetic Improvement, Ecology and Physiology, Jinan 250100, PR China

## Abstract

Different from most of other plants, peanut (*Arachis hypogaea* L.) is a typical geocarpic species which flowering and forming pegs (gynophores) above the ground. Pegs penetrate into soil for embryo and pod development. To investigate the molecular mechanism of geocarpy feature of peanut, the proteome profiles of aerial grown gynophores (S1), subterranean unswollen gynophores (S2), and gynophores that had just started to swell into pods (S3) were analyzed by combining 1 DE with nano LC–MS/MS approaches. The proteomic data provided valuable information for understanding pod development of peanut. The data described here can be found in the PRIDE Archive using the reference number PXD002579-81. A more comprehensive analysis of this data may be obtained from the article in BMC Plant Biology (Zhao et al., 2015 [1]).

**Specifications table**

**Table:** 

Subject area	*Biology*
More specific subject area	*Plant development*
Type of data	*Table, figure and excel file*
How data was acquired	*The nano HPLC system (Shimadzu, Kyoto, Japan)*
*Tandem mass spectrometry (MS/MS) of LTQ Orbitrap Velos (Thermo)*
Data format	*Raw data*
Experimental factors	*Samples were collected from the farm-grown plants under normal conditions*
Experimental features	*Specific and common proteins in aerial gynophores, subterranean unswollen gynophores, and early swelling gynophores were analyzed.*
Data source location	*Ji’nan, China*
Data accessibility	*All data are available with this article. The proteomics raw data files can be found at ProteomeXchange with identifier PXD002579-81.*

**Value of the data**

• The data reported thousands of proteins in three stages of peanut pod development, which providing the comprehensive proteome data to identify potential proteins in the peanut. And the data can also be used as a resource in peanut proteome comparative research.
• The data demonstrated the specific or highly abundant proteins in the aerial gynophores, subterranean gynophores, and early swelling pods of peanut, which lays the foundation for understanding the geocarpy mechanisms of peanut at the protein level.
• The data identified 69 proteins involved in the gravity response, light and mechanical stimulus, hormone biosynthesis, and transport, suggesting the important roles of light, mechanical stimulus and hormone in peanut pod development.

## 1 Data, experimental design, materials and methods

**Fig. 1:**
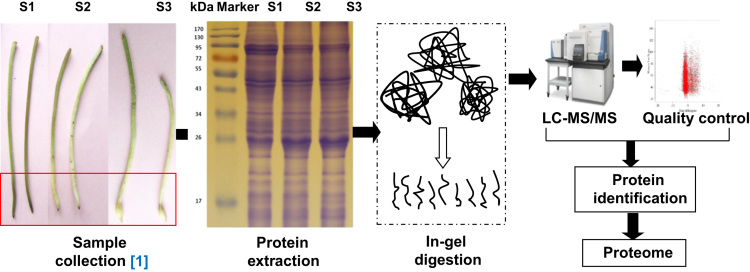
Workflow employed to characterize the peanut proteome.

The data in the PRIDE Archive provide a comprehensive protein identification of different stages of peanut gynophores [2]. This data can be analyzed using a variety of commercial tandem mass spectrometry tools. The data provide variable information for researchers to study the functions of potential key genes in peanut pod development. The following sections present a detail description about the materials and methods, will help the investigators to design novel procedures that rely on 1 DE with nano LC–MS/MS approaches (Fig. 1).

### 1.1 Sample preparation

Cultivated peanut Luhua14 was planted in the experimental farm under normal conditions. The aerial gynophores that are green or purple and 3–5 cm long, the white unswollen gynophores that have been buried in the soil for about 3 days, and dark-grown gynophores with 2–3 mm long pods were collected (Fig. 1).

### 1.2 Protein separation and In-gel digestion

The procedure of protein separation and digestion was based on the approaches previously reported [3], [4]. Briefly, proteins were extracted with lysis buffer 1 (7 M Urea, 2 M Thiourea, 4% CHAPS, 40 mM Tris–HCl, 1 mM PMSF and 2 mM EDTA, pH 8.5) and then mixed with 10 mM DTT (final concentration) for 5 min. Then samples were disrupted by sonication at 200 W for 15 min and then centrifuged at 30,000*g* for 15 min. After centrifugation, the samples were mixed with 5×volume of chilled acetone containing 10% (v/v) TCA and incubated overnight. The precipitate was obtained by centrifugation at 30,000*g* for 15 min. Then they were washed, dissolved with lysis buffer 2 (7 M urea, 2 M Thiourea, 4% NP40, 20 mM Tris–HCl, pH 8.0–8.5), and disrupted with sonication again. The supernatant was collected by centrifugation. Each protein concentration was determined using a 2-D Quant Kit.

Proteins were separated by 12% polyacrylamide gel electrophoresis. The gel with protein bands was vertically cut into 10 slices, each gel slice was destained with 50 mM ammonium bicarbonate in 50% ACN, and then was incubated in 10 mM DTT with 25 mM ammonium bicarbonate to reduce disulfide bonds. Alkylation of cysteines was performed by incubating the samples with 55 mM iodoacetamide in 25 mM ammonium bicarbonate for 45 min at room temperature in dark. After digested with Trypsin Gold, the peptides were extracted from gel bands using 0.1% formic acid in 50% ACN twice.

### 1.3 Peptide fractionation and LC–ESI–MS/MS analysis

The dried peptide samples were reconstituted with 4 ml buffer A (25 mM NaH_2_PO_4_ in 25% ACN, pH 2.7) and separated by SCX Chromatograhpy (Shimadzu, Kyoto, Japan), which equipped with a 4.6×250 mm C-18 HPLC column. Before the sample was injected into the column, the column was previously equilibrated with buffer A for 10 min. The elution procedure was designed as follows: first buffer A washed for 10 min, then 5–60% buffer B (25 mM NaH_2_PO_4_, 1 M KCl in 25% ACN, pH 2.7) washed for 27 min, and then 60–100% buffer B for 1 min, and 100% buffer B washed for 1 min before the next separation. The flow rate was 1 ml/min. The peptides were monitored by measuring the absorbance at 214 nm, and fractions were collected per 1 min. The peptides were pooled into 20 fractions, desalted with Strata X C18 column (Phenomenex) and vacuum-dried.

In order to remove the insoluble materials, the digested peptide samples were redissolved in buffer A containing 5% ACN and 0.1% FA, and then centrifuged for 10 min. The supernatant was separated using HPLC chromatography (Shimadzu, Japan) following the instructions.10 µl of sample was injected into a C18 trapping column (10 cm, inner diameter, 75 μm; Waters, USA ) at a flow rate of 8 μl/min for 4 min, and the elution procedure was determined as follows: 2–35% buffer B (98% ACN and 0.1% FA) washed for 44-min, 35–80% buffer B washed for 2 min, 80% buffer B washed for 4 min, 5% buffer B washed for 1 min, with a flow rate of 300 μl/min. Then, the separated peptides were subjected to tandem mass spectrometry (MS/MS) in a LTQ Orbitrap Velos (Thermo, USA) coupled online to the HPLC system. Intact peptides were scanned in the Orbitrap at a resolution of 60,000, and further selected for collision-induced dissociation (CID) operating with a normalized collision energy setting of 35%. The ion fragments were detected in the LTQ. A data-dependent procedure that alternated between one MS scan followed by 10 MS/MS scans was applied, and the 10 most intense ions above a threshold ion count of 5000 were detected with the following Dynamic Exclusion settings: repeat counts, 2; repeat duration, 30 s; and exclusion duration, 120 s. The applied electrospray voltage was 1.5 kV. Automatic gain control (AGC) was used to prevent overfilling of the ion trap and 1×104 ions were accumulated in the ion trap to generate CID spectra. For the MS scans, the *m*/*z* scan range was 350–2000 Da.

### 1.4 Data analysis and statistics

Raw data files obtained from the Orbitrap were converted into MGF files using Proteome Discoverer 1.2 (Thermo, USA). All raw data acquired on the LTQ-Orbitrap is available in the PRIDE Repository (http://www.ebi.ac.uk/pride/archive/projects/PXD002579-81). Protein annotation was performed using the Mascot search engine (Matrix Science, UK; version 2.3.02) against the peanut transcriptome database, which contained 72,527 unigenes [5]. The false discovery rate (FDR) was set to 0.05 for both peptides and proteins. Overall, 2766, 2518, and 2280 proteins were identified from three samples, respectively [1]. These proteomics data identified the common and specific proteins in different stages of peanut gynophore development, revealed the candidate genes involved in geocarpy of peanut.
